# Japanese Encephalitis: a case of remarkable recovery after 68 days of ICU admission in a 30 -years-old-male

**DOI:** 10.1186/s12879-026-12540-2

**Published:** 2026-01-12

**Authors:** Bishal Budha, Ziyaul Haq Musalman, Sabin Thapaliya, Roshan Chaudhary, Dhiraj Adhikari, Sushil Sah, Dipesh Regmi

**Affiliations:** 1 Maharajgunj Medical Campus, Institute of Medicine, Tribhuvan University Teaching Hospital, Maharajgunj, Kathmandu 44600 Nepal; 2 Department of Internal Medicine (Infectious Diseases), Tribhuvan University Teaching Hospital, Maharajgunj, Kathmandu Nepal

**Keywords:** Japanese Encephalitis, Culex mosquito, Neurological sequelae, Neurological recovery, Case report

## Abstract

**Introduction and importance:**

Japanese Encephalitis is a disease of central nervous system caused by Japanese Encephalitis Virus (JEV), a flavi virus transmitted by culex mosquitoes. Only < 1% of the infected people develop symptoms and among symptomatic cases, around 1/3rd die and remaining 30–50% develop persisted neurological damage. Here, we present an unusual case of 30 years male with severe neurological manifestations requiring intubation and ICU admission for 68 days but miraculously recovering completely without any residual neurological deficits.

**Case presentation:**

We present a case of 30 years Male who presented with high grade fever, headache, vomiting which progressed to altered sensorium and decreased GCS requiring intubation. CNS infection was suspected based on presentation and diagnosis of JE was confirmed with CSF anti-JEV IgM ELISA however CNS imaging was not significant. He was managed in ICU for 68 days with supportive care, antibiotics for ventilator associated pneumonia and physiotherapy. On long term follow up on outpatient basis, he unexpectedly recovered fully with no neurological deficits despite severe manifestations on initial presentation.

**Discussion:**

Japanese Encephalitis, a disease of the CNS, is often asymptomatic, but once a patient becomes symptomatic, it has very bad prognosis with high morbidity and mortality and many patients end up developing lifelong neurological deficits. Our case says otherwise, in which our patient undergoes complete recovery despite severe manifestations on initial presentation. Although this is not the only reported case of recovery from Japanese Encephalitis with severe manifestations at presentation, complete recovery is very rare and this case emphasizes on favorable factors and timely intervention that can help reduce morbidity and mortality in similar cases in the future.

**Conclusion:**

This case presents an unusual outcome of JE. Although the prognosis of JE isn’t great, from this case we can say that despite severe presentation we can have favorable outcomes with supportive care and long-term physiotherapy. It’ll also be helpful while counseling the patient and patient parties on the prognosis of JE. Further research is required to update the outcomes in JE patients in current healthcare settings.

**Supplementary Information:**

The online version contains supplementary material available at 10.1186/s12879-026-12540-2.

## Introduction

Japanese Encephalitis is a zoonotic infection caused by Japanese Encephalitis Virus (JEV), a flavivirus transmitted by mosquitoes, principally *Culex tritaeniorhynchus.* [1] It is endemic in south east Asia and around 51.9% population of Nepal are living in at risk areas [2]. It is more prevalent in regions where Culex mosquitoes breed, particularly in areas with paddy fields and standing water. Most patients who are infected remain asymptomatic. Only < 1% people develop flu like symptoms and among symptomatic cases around 1 in 250 develop severe disease; 33% die and around 30–50% develop persistent neurological damage [3]. Clinical features varies widely from febrile illness associated with headache to more severe manifestations like confusion, convulsions, neurological deficits which are often persistent, coma and death [4, 5]. 

Here, we present a case of Japanese Encephalitis presenting with severe manifestation requiring 68 days of ICU admission but having full recovery over long term follow up and supportive care unlike usual cases.

## Case presentation

A 30-year-old male from rural Nepal, with rice cultivation and livestock farming nearby, presented to the Emergency department of a local hospital with a four days history of high-grade fever reaching 39 °C, associated with chills and rigor, multiple episodes of vomiting per day, and generalized, moderate-to-severe headache. On the fifth day of illness, he developed slurred speech and progressive decrease in responsiveness. There were no features of meningism, or any gastrointestinal or respiratory complaints. He had a 3.5 pack year of smoking history but no history of Diabetes, Hypertension or any chronic illness. Family history and allergy history was unremarkable. Due to deteriorating sensorium, he was intubated for airway protection following a decline in GCS after fifth day and was transferred to the ICU of nearby higher center, the very bext day where he was admitted for 20 days. Subsequently, he was referred to our tertiary care center for further evaluation and management.

### Diagnostic assessment

On initial assessment at our center, he was ill looking, with a tracheostomy tube in situ. His vital signs were: BP 140/100 mmHg, RR 20/minute, and HR 100 bpm. GCS was E_4_V_T_M_6_. Neurological examination was evident for asymmetrically decreased strength in all extremities; (MRC 3/5) in the right upper extremity and (4/5) in all other extremities. Reflexes were 2 + and Babinski was down going. He was able to follow commands. Sensation was intact and he had no signs of meningism or abnormal body movements. Pupils were round, regular and reactive to light. Systemic examination was unremarkable. He was admitted to ICU for further management.

Before referring to our center, he had already had 25 days of hospital admission with 20 days of ICU care. He had been diagnosed with Japanese Encephalitis based on CSF findings: Anti-JE IgM positive, cell count 200/mm^3^ (predominantly lymphocytes), glucose 62 mg/dl and protein 80 mg/dl on twelfth day of disease onset. A non-contrast CT scan of Head was normal. Subsequent contrast-enhanced MRI Brain and Cervical spine was done which revealed few discrete and confluent T2 and FLAIR hyperintense foci in B/L periventricular white matter and centrum semiovale, likely non-specific foci of demyelination.

During the hospital course, he developed hospital acquired pneumonia for which sputum culture and sensitivity was sent. *Pseudomonas aeruginosa* and *Klebsiella pneumoniae* were isolated sensitive to Meropenem and Piperacillin-tazobactum.

### Diagnostic reasoning

Viral encephalitis was suspected based on initial presentation of high fever, headache, decreased responsiveness and motor deficits and the biochemical picture of CSF analysis which showed increased cells (predominantly lymphocytes), normal glucose and slightly increased protein. Given the epidemiological setting, Japanese Encephalitis (JE) was strongly considered, and the diagnosis was confirmed by detection of anti-JEV IgM antibody in CSF using ELISA, which also excluded other flavi-viral infections. Neuroimaging was performed to localize the site and extent of involvement and to rule out other etiologies like brain abscess, tumor, other viral encephalitis like HSV, EBV, CMV etc. PCR testing was not pursued due to financial constraints and the already established diagnosis of JE through ELISA.

### Intervention

At the referring center, he was empirically treated for suspected meningitis with intravenous fluids and broad spectrum antibiotics (Ceftriaxone 2gm twice daily and vancomycin 1gm twice daily). These were discontinued once CSF analysis confirmed JE. He required intubation for progressive worsening of consciousness, which was subsequently converted to tracheostomy on day 10. He remained in ICU for a total of 20 days before being transferred to our center. During his stay, he developed ventilator associated pneumonia which was managed with antibiotics.

At our tertiary center, he was co-managed by Neurology and Infectious Disease team and admitted to ICU for total of 68 days. Management included intravenous fluids, multiple courses of antibiotics tailored to culture sensitivity (meropenem, levofloxacin, linezolid, doxycycline) for VAP, which gradually resolved, and Lacosamide (50 mg) for seizure prophylaxis. Hypertension was managed with amlodipine, losartan, and clonidine, along with other supportive measures. The tracheostomy tube was progressively downsized and eventually removed on 63^rd +^ tertiary centered ICU admission. Physiotherapy was initiated during hospitalization and continued on an outpatient basis following discharge.

**Fig. 1:**
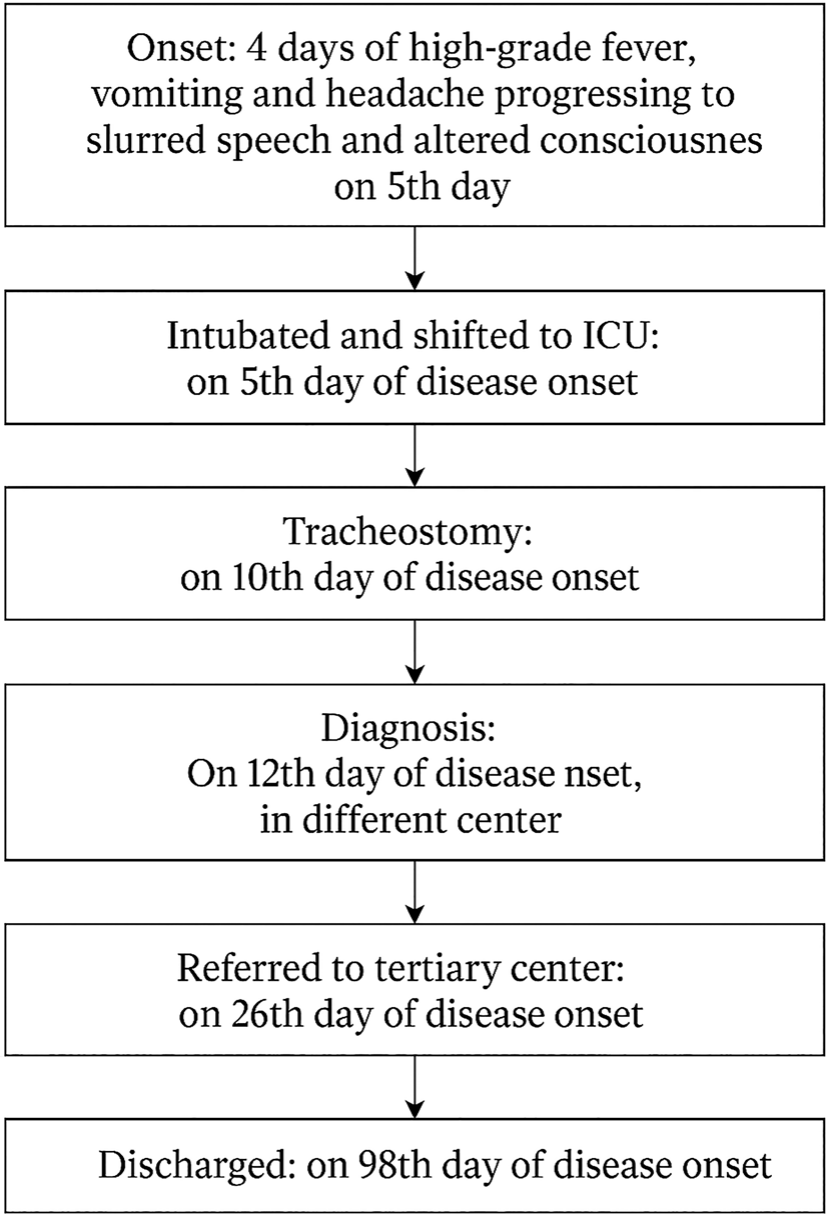
Flowchart of disease progression

He was then followed up monthly for the first three months and subsequently at three-months intervals. At his most recent follow-up visit, 9 months after discharge, clinical examination revealed no residual neurologial deficits, with complete recovery of motor power in both upper and lower limbs. He reported no recurrent seizures, cognitive impairment, or other neurological complaints. Overall, he demonstrated full resolution of symptoms, indicating complete recovery from Japanese Encephalitis (Fig 1).

## Discussion

Japanese Encephalitis(JE) is an inflammatory disease of Central Nervous System(CNS) caused by JEV, a flavivirus and is the most common cause of viral encephalitis in Asia [6]. It is a mosquito borne zoonotic infection and uses *Culex tritaeniorhynchus* as a vector for transmission which is common in paddy fields and water logged areas [7]. WHO estimates around 68,000 annual cases worldwide and it’s a major public health concern in Nepal [7]. The number of cases has significantly dropped after introduction of vaccine against JEV which is 95 to 99% effective [7]. Immunity starts getting weakened at around 25 years of age that’s why JE infection is getting more common in middle aged population now [3]. 

Only 0.1-1% of infected develop encephalitis and majority remain asymptomatic but still it remains a neurological disease with highest global burden due to its long term neurological sequelae [8]. Patient commonly presents fever, coryza, myalgias which then progresses to headache, vomiting, altered sensorium and seizures. Important neurological manifestation include generalized tonic-clonic seizures, focal seizures, parkinsonian syndromes, cranial nerve palsies, acute flaccid paralysis and behavioral abnormalities [8]. Our patient also presented with similar symptoms like fever, persistent headache, vomiting and later he had decreased responsiveness and dropping GCS requiring intubation. That’s why clinical suspicion of JE was high especially considering the epidemiological background.

Detection of Anti JEV IgM antibodies using ELISA plays crucial role in diagnosis of JE. It should be tested in both blood and CSF but CSF one is more specific. In cases where CSF sample can’t be taken, diagnosis can be made using clinical and imaging features along with positive serum anti JEV IgM antibodies [3]. On early presentation, antibodies may be negative and should be repeated at a later date if clinical suspicion is high. It can also differentiate other flavivirus infections. Imaging of brain shows characteristic focal lesions in unilateral or bilateral thalamus, basal ganglia, brainstem which are hypodense in CT and hyperintense in T2 weighted imaging (T2WI) and FLAIR signals in MRI and in some cases obvious brain edema is seen [3]. MRI is more sensitive than CT scan. CSF PCR can also be done to detect or rule out other viruses like HSV, EBV, CMV etc. In our patient as well, CSF analysis was done which showed increased protein and cells (predominantly lymphocytes) and normal glucose which is typical of viral encephalitis. CSF anti JEV IgM was positive and thus the diagnosis was made. CT scan was normal and MRI showed nonspecific foci of demyelination. PCR was not done due to high cost and serum anti JEV IgM was not sent as CSF result already came out to be positive.

There’s no specific treatment of JE. Treating high fever, raised ICP, seizures, preventing and managing complications are the mainstay of therapy [3]. Our patient was also initially empirically managed with acyclovir and Ceftriaxone but antibiotics were stopped after diagnosis of JE was made. Due to his poor GCS score (7/15), he was intubated and later tracheostomy was done on 10th day. He stayed in ICU for a total of 68 days during which he received supportive care, anti- seizure medications prophylactically, multiple antibiotics to treat ventilator associated pneumonia and antihypertensive for controlling high blood pressure. Physiotherapy was started early in ICU on and was continued over long term in outpatient visits. Tracheostomy was downsized and strapping was done before he was discharged.

Around 30% of the symptomatic cases die and among the survivors 50% developed long term persistent neurological sequelae like frank motor deficits, fixed flexon deformity of arms, equine feet, cognitive and language impairment etc [9]. This case particularly highlights the remarkable recovery of the patient despite severe manifestations and we attribute this favorable outcome to several key factors. Patient’s young age and absence of co morbidities allowed a robust immune response and greater potential for neurologic recovery. Early and aggressive supportive care including initiation of a structured physiotherapy regimen was pivotal in this case that likely helped prevent complications. This case also highlights the importance of multi-disciplinary approach for long term functional restoration.

### Learning points

1. Clinicians need to maintain a proactive and optimistic approach to management of JE especially in young patients.
2. Prognosis is multifactorial and focus should be on modifiable factors including timely and aggressive intervention.
3. Physiotherapy should not be viewed as a secondary intervention but as am integral component of acute management.
4. Patient adherence is critical and complex sequale of JE demands a multi-disciplinary approach.

## Conclusion

Our case underscores that, although Japanese Encephalitis carries a poor prognosis and may present with severe illness necessitating intubation and prolonged ICU care, a favorable outcome is still possible. Despite developing severe neurological manifestations, our patient achieved complete recovery through supportive care, physiotherapy, and meticulous management of complications in a resource-limited setting. This emphasizes the need for cautious optimism when counseling patients and families regarding JE outcomes. However, further research is necessary to clarify long-term prognoses in JE patients at current healthcare environments.

## Supplementary Information

Below is the link to the electronic supplementary material.

Supplementary Material 1

## Data Availability

No datasets were generated or analysed during the current study.

## Abbreviations

JE: Japanese encephalitis
CSF: Cerebrospinal fluid
ICU: Intensive care unit
CT: Computed tomography
MRI: Magnetic resonance imaging
CNS: Central nerve system
