# Supplementary material for: Japanese Encephalitis: a case of remarkable recovery after 68 days of ICU admission in a 30 -years-old-male
Source: BMC Infect Dis. 2026 Jan 12;26:293. doi: 10.1186/s12879-026-12540-2 (PMC12888258; doi:10.1186/s12879-026-12540-2)
Supplement: Supplementary file 1 — Supplementary Material 1 [file 12879_2026_12540_MOESM1_ESM.pdf]

CARE Checklist of information to include when writing a case report

Topic	Item	Checklist item description	Reported on Line
**Title**	**1**	The diagnosis or intervention of primary focus followed by the words “case report”	**page 1**

CARE
case report guidelines

(cc) BY-NC-ND

✓

Key Words 2 2 to 5 key words that identify diagnoses or interventions in this case report, including "case report" .page 1

Abstract 3a Introduction: What is unique about this case and what does it add to the scientific literature? . . .**page 1**
. . . .

(no references) **3b** Main symptoms and/or important clinical findings . . . . **page 1. . . . .**
. . . .

3c The main diagnoses, therapeutic interventions, and outcomes . . . .**page 1 . . . .**
. . . .

3d Conclusion—What is the main “take-away” lesson(s) from this case? . . . . **page 1. . . . .**
. . . .

Clinical Findings

Timeline

Diagnostic Assessment

Introduction 4 One or two paragraphs summarizing why this case is unique (**may include** reference**s**) . . . . .**page 2 . . . .**

Patient Information **5a** De-identified patient specific information. . . . .
. . . .

5b Primary concerns and symptoms of the patient. . . . . . .**page 3 . . . .**
. . . .

Therapeutic Intervention

5c Medical, family, and psycho-social history including relevant genetic information . . . .

5d Relevant past interventions with outcomes . . . . **page 3. . . . .**
. . . .

Follow-up and Outcomes

6 Describe significant physical examination (PE) and important clinical findings. . . . .**page 2 and 3** . . .
. . . .

7 Historical and current information from this episode of care organized as a timeline . . . .
. **8a** Diagnostic testing (such as PE, laboratory testing, imaging, surveys). . . . .**page 3** . . . .
. . . .

Discussion

8b Diagnostic challenges (such as access to testing, financial, or cultural) . . . . **page 2 and 3. . . . .**
. . . .

8c Diagnosis (including other diagnoses considered) . . . .**page 3 . . . .**
. . . .

Patient Perspective

Informed Consent

8d Prognosis (such as staging in oncology) where applicable . . . . **. page 2 and 3. . . . .**
. . . .

9a Types of therapeutic intervention (such as pharmacologic, surgical, preventive, self-care) . . . . . .**page 3** ☐ **No** ☐
. . . .

9b Administration of therapeutic intervention (such as dosage, strength, duration) . . . . **page 2 and 3.** . . . . . .
. . . .

9c Changes in therapeutic intervention (with rationale) . . . . **page 3.** . . . .

10a Clinician and patient-assessed outcomes (if available) . . . . **page 3.** . . . .

10b Important follow-up diagnostic and other test results . . . . **.page 3** . . . .

10c Intervention adherence and tolerability (How was this assessed?) . . . . **.page 3** . . . .

10d Adverse and unanticipated events . . . . **.page 3** . . . .

11a A scientific discussion of the strengths AND limitations associated with this case report . . . . . **.page 4** . . . .

11b Discussion of the relevant medical literature **with references**. . . . . **.page 5** . . . .

11c The scientific rationale for any conclusions (including assessment of possible causes) . . . **.page 4** . . . .

11d The primary “take-away” lessons of this case report (without references) in a one paragraph conclusion .page4. .

12 The patient should share their perspective in one to two paragraphs on the treatment(s) they received . . . .

13 Did the patient give informed consent? Please provide if requested . . . . **Yes**
